# Clinical Outcome After Surgery for Fracture-Related Infection Is Dependent on Both Microbiology and the Host Inflammatory Response

**DOI:** 10.3390/pathogens15050532

**Published:** 2026-05-14

**Authors:** Ruth A. Corrigan, Andrew J. Hotchen, Anton A. N. Peterlin, Louise K. Jensen, Martin McNally

**Affiliations:** 1 The Bone Infection Unit, Nuffield Orthopaedic Centre, Oxford University Hospitals, Oxford OX3 9DU, UK; ruth.corrigan@ouh.nhs.uk (R.A.C.); andrew.hotchen@ouh.nhs.uk (A.J.H.); 2 Nuffield Department of Orthopaedics, Rheumatology and Musculoskeletal Medicine, University of Oxford, Oxford OX1 2JD, UK; 3 Department of Orthopaedic Surgery, Herlev Hospital, 2730 Herlev, Denmark; anton.peterlin@sund.ku.dk; 4 Department of Veterinary and Animal Sciences, University of Copenhagen, 1165 København, Denmark; louise-k@sund.ku.dk

**Keywords:** fracture-related infection, FRI, microbiology, histology, clinical outcome, treatment, osteoimmunology, immune response

## Abstract

Microbiological culture and histology of deep tissue specimens are independent diagnostic criteria in fracture-related infection (FRI). However, the association between these tests has rarely been investigated, particularly in relation to clinical outcome after treatment. Patients undergoing surgery for International Consensus-confirmed FRI were included. All had ≥5 tissue specimens taken for microbiological culture and 2–3 for histology. The correlation between cultured pathogen, histological positivity (defined as ≥5 polymorphonuclear neutrophils/high power field), and outcome at one year after surgery was explored. FRI was confirmed in 430 patients, predominantly in the tibia (194), femur (111), upper limb (70), and ankle (40). A total of 321 (74.7%) were culture-positive and 334 (77.7%) were histology positive, while 265 (61.6%) were positive for both tests. *Staphylococcus aureus* was cultured in 169 (42.5%), coagulase-negative Staphylococci (CoNS) in 61 (15.3%), and Gram-negatives in 145 (36.3%) cases. Virulent microorganisms were strongly associated with positive histology (odds ratio 2.72; 95% CI 1.61–4.58) but not with clinical failure (OR 1.08; 0.42–2.75). Isolation of *S. aureus* was significantly associated with positive histology compared to other microorganisms (OR 2.21; 1.27–3.87). Surgery succeeded in 390 (90.7%) patients. Treatment failure was weakly associated with positive microbiology alone (OR 2.03; 0.83–4.96) or positive histology alone (OR 2.13; 0.81–5.6). Combined positive culture and histology was strongly associated with clinical failure (OR 2.3; 1.06–4.96). There was no difference in outcome between virulent and non-virulent bacteria when histology was positive, but both had higher failure rates compared to patients with negative culture or histology. A pronounced inflammatory response, as seen in histology, is a feature of virulent bacterial FRI. However, the presence of virulent infection alone does not dictate clinical outcome without marked inflammation. This suggests that outcome is at least as much related to the host response as to the bacterium. When the pathological response is prominent, this may lead to tissue necrosis, further bacterial invasion of adjacent tissues, osteolysis and loss of fracture stability, contributing to treatment failure. This deserves further study to understand the mechanisms behind this interplay and clinical outcome.

## 1. Introduction

Fracture-related infection (FRI) is a significant cause of fracture non-union and patient morbidity and mortality [1,2,3]. Infection may affect 30–40% of open fractures due to direct contamination and soft tissue injury [4,5,6], but the rate remains as high as 1–2% for closed fractures, likely due to pathogen inoculation during surgery to stabilize the fracture.

FRI begins with ingress of bacteria into the fracture site. These bacteria may elicit a variable inflammatory response, depending on the presence or absence of virulence factors, time to immune cell acknowledgment, the degree of tissue invasion, and tissue injury. When the pathological response is severe, this may lead to tissue necrosis, further invasion of adjacent tissues, fibrosis, and osteolysis, resulting in loss of fracture stability [7]. Altogether, such a pathological response inhibits osteogenesis, causing failure of fracture union. Proposed pathophysiological mechanisms include both neutrophil-released proteolytic enzymes, direct pathogen-mediated bone destruction, and pathogen persistence, which stimulates fibroblast proliferation and thus fibrosis formation [8,9].

Recent consensus definitions of FRI include either positive microbiology or positive histology (defined as ≥5 polymorphonuclear neutrophils/high power field, as a proxy measure of inflammation) as independent diagnostic criteria, acknowledging that patchy or intracellular infection or recent antimicrobial use may render samples culture-negative while the associated inflammation remains [10,11].

Furthermore, it is increasingly recognized that antibiotic pressure may render bacteria in a viable but non-culturable state, often associated with biofilm-related infections [12,13,14], and that *Staphylococcus aureus* may persist inside host cells, including macrophages, osteoblasts, osteoclasts, and osteocytes [8,15]. It is hypothesized that the presence of intracellular bacteria may even subvert the function of bone building osteoblasts, in addition to direct osteoclast stimulation via RANKL [16]. Regarding macrophages, intracellular persistence promotes M2 differentiation, a state associated with immunosuppression and sustained chronic infection [17,18,19]. It has also been shown that *Staphylococcus aureus* can invade and persist as small colony variants within the canaliculi of bone, where they resist inhibitory concentrations of antibiotics [20,21,22].

Bone infection is increasingly understood to be an osteoimmunological process rather than colonization of injured tissue. The innate immune cells that are recruited to bone following the ingress of bacteria can amplify local tissue injury and disrupt bone repair [23]. Inflammatory mediators such as TNF-α, Interleukin (IL)-1, IL-6, and RANKL can promote osteoclast differentiation and activity, while inflammatory stress can impair osteoblast function and osteogenesis [24]. Furthermore, osteocytes can directly sense pathogen-associated molecular patterns and drive RANKL-mediated osteolysis [24]. This provides a biological basis for the hypothesis that the histological inflammatory status may be linked to outcome in FRI.

Increasingly, transcriptomics is being utilized to identify virulence factors of the pathogens implicated in bone infections. Studies have found differential expression of virulence factors involved in metabolism, proteolysis of host proteins, and immune evasion between *Staphylococcus aureus* isolates in acute and chronic infection [5] and that genes associated with antigen presentation and apoptosis are differentially expressed in hosts with osteomyelitis versus those without [6].

One recent study found a trend towards virulent pathogens, defined as uncommon contaminants, such as *Staphylococcus aureus* and Gram-negatives [25,26], more often being associated with prominent inflammation on histology in FRI and osteomyelitis [27]. However, this study was too small to allow a robust assessment, particularly with regard to clinical outcome. The larger study by Achatz et al. (2026) suggested that in prosthetic joint infection, failure of treatment was much more frequent with highly virulent Gram-positive and Gram-negative microorganisms [28].

This study aimed to clarify the relationship between microbiology, inflammation, and clinical outcome after surgical treatment with respect to fracture-related infection.

## 2. Materials and Methods

Patients treated surgically for FRI of the appendicular skeleton between 1 January 2015 and 31 December 2019 at the Bone Infection Unit, Nuffield Orthopaedic Centre, Oxford, UK, were retrospectively evaluated. FRI was defined according to the criteria of the International FRI Consensus definition [10,11]. Patients were eligible for inclusion if they received definitive operative treatment for FRI, as defined by the treating surgeon. Minimum follow-up was at least one year with documentation of clinical outcome. For all patients, antibiotic therapy had been stopped at least two weeks before sampling.

All patients had at least 5 deep tissue specimens taken from the fracture site, with separate instruments, for microbiological culture [29,30]. Positive microbiology and the causative pathogen were defined as (1) isolation of phenotypically identical microorganisms from two or more surgically obtained deep tissue specimens [10,30] or (2) when the definition of FRI was met based on non-microbiological criteria. Isolation of a single uncommon contaminant (a virulent pathogen) from a single deep tissue specimen was accepted as a positive microbial culture [25]. In the literature, pathogens have been categorized by their differences in expression of virulence factors such as toxin production and biofilm formation [26]. Numerous studies have agreed on which organism can be considered as virulent [25,28,31,32,33]. In contrast, single isolates of *Staphylococcus epidermidis* and *Corynebacterium* species, for example, were considered contaminants when this was the only positive microbiology. When no pathogens were isolated, the case was considered culture-negative. In polymicrobial infections, microbiology was classed as virulent if at least one virulent pathogen was isolated.

Intraoperative samples were collected in sterile universal containers, pre-filled with 3 mL saline and sterile glass beads (Equine and Ovine Laboratories). Samples were disrupted by vortexing at 40 Hz for 15 s. Any metalwork removed was placed in a sonication container and covered with sterile saline to at least 90% of its volume. Containers were vortexed for 30 s, sonicated in an ultrasound bath for 1 min and vortexed again for 30 s. An amount of 0.5 mL of each sample was inoculated into a BD BACTEC™ Lytic 10 Anaerobic/F bottle and 0.5 mL was inoculated into a BD BACTEC™ Lytic 10 Aerobic/F bottle (Becton, Dickinson and Company, Franklin Lakes, NJ, USA). Culture bottles were incubated for up to 10 days at 37 °C and any that flagged positive were sub-cultured on agar [34,35].

**Figure 1:**
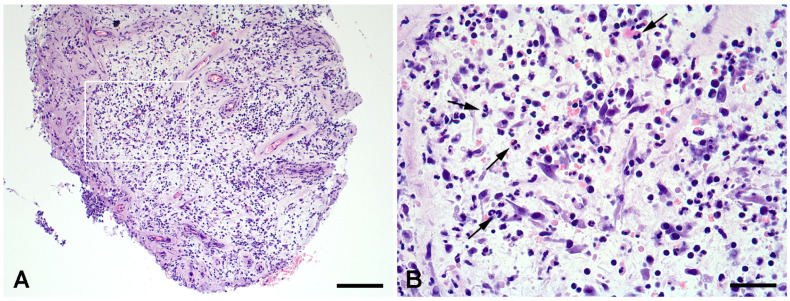
Histology of a *S. aureus*-positive FRI patient. (**A**) Overview of debridement biopsy showing granulation tissue with infiltration of inflammatory cells. Hematoxylin & eosin, magnification ×20 (scale bar 200 μm). (**B**) High-power field of A (white box) showing numerous neutrophils (black arrows) (>5 PMNs/HPF). Magnification ×400 (scale bar 50 μm).

Two or three tissue specimens were taken from the same areas as the microbiology samples for histological analysis. All samples were fixed in formalin and paraffin-embedded. Sections of 5 μm were cut and stained with hematoxylin and eosin (H&E). Sections were initially examined under low power to identify areas of maximum inflammation. In these inflamed areas, at least 10 high power fields (×400 magnification) were examined and the number of neutrophils was counted. Positive histology was defined as a mean of ≥5 neutrophils per high power field at 400× magnification, averaged across the ten fields examined [36] (Figure 1). The reviewing pathologist was unaware of the microbiological or clinical outcome of the patient.

Treatment failure was defined as previously published [25]. Namely, it included recurrence of infection (fulfilling FRI definition), unplanned surgery outside the period of the definitive surgery for infection or skin coverage, an unhealed wound or discharge from the surgical site beyond three months after the period of definitive surgery, and commencement of further antibiotic therapy for infection related to the surgical site (other than for external fixator pin site infection) after the initial planned antibiotic regimen was discontinued.

Statistical analysis was performed using SPSS v29.0 (IBM Corp., Armonk, NY, USA). Odds ratios were calculated to compare relationships between tests and outcome, with 95% confidence intervals. *p* < 0.05 was regarded as statistically significant.

## 3. Results

In total, 444 FRIs were reviewed. A total of 14 had no histology specimens taken and so were excluded. Microbiology results were available for the remaining 430 cases included in this study. Of those included, 321 (74.7%) had positive microbiology, and the remainder were classified as culture-negative. Of the culture-positive cases, 229 (53.4%) patients were monomicrobial, and 92 (21.4%) were polymicrobial. A total of 334 cases were histology positive (77.7%) and 265 (61.6%) were positive for both tests.

### 3.1. Demographics, Infection Type and Causative Pathogens

The mean patient age at surgery was 51.7 years, and 108 (25.1%) of patients were female. Infection predominantly affected the tibia (194, 45.0%), followed by the femur (111, 25.8%), upper limb (70, 16.2%) and ankle (40, 9.3%). A total of 484 pathogens were isolated from the 430 cases. A total of 390 of these were considered virulent, versus 94 non-virulent. *Staphylococcus aureus* was the predominant pathogen, isolated in 169 cases (42.5%), with CoNS isolated in 61 cases (15.3%) and Gram-negatives in 145 (36.3%) cases (Table 1 and Table 2).

### 3.2. Association of Histology with Virulent and Non-Virulent Organisms

Histology was more likely to be positive when the identified bacteria included at least one virulent pathogen, compared to only non-virulent pathogens (86.2% of FRIs vs. 69.1%; OR 2.72 (95% CI: 1.61–4.58)). Of note, histology positivity was independent of the number of infecting species, occurring in 82.5% of monomicrobial FRIs and 82.6% of polymicrobial FRIs (OR 0.995 (95% CI: 0.53–1.88)). Similarly, there was no difference in histology positivity if the isolated microorganisms were only Gram-negative or only Gram-positive or mixed Gram-positive and Gram-negative (83.0% vs. 82.6% vs. 81.4%; Χ^2^ = 0.0796, *p* = 0.961).

By pathogen, *Staphylococcus aureus* was most strongly associated with positive histology compared to all other organisms (OR 2.21; 1.27–3.87) and when compared with other Gram-positive organisms (OR 2.7; 1.48–4.95) (Table 2).

### 3.3. Associations of Histology and Microbiology with Outcome

Failure of surgical treatment was more frequent when microbiological culture was positive, virulent organisms were present, or histology was positive. Failure was much more likely with polymicrobial infection compared to monomicrobial and to culture-negative cases. Failure rate was highest with mixed Gram-positive and Gram-negative polymicrobial infection (18.6%). Failure was less than half as frequent with monomicrobial infection with either Gram-positive or Gram-negative microorganisms alone (OR 0.42; 0.19–0.92).

However, overall clinical failure rate was not statistically significantly associated with either positive or negative microbiology in isolation (OR 2.03 (95% CI; 0.83–4.96)). The presence of virulent microorganisms alone did not dictate clinical failure, compared to non-virulent infection (10.2% vs. 10.9%) (OR 1.08; 0.42–2.75). Also, the high failure rate in mixed Gram-positive and Gram-negative FRIs was entirely due to those with positive histology, with no failures in the histology negative patients (22.9% vs. 0%). Similarly, clinical failure was not significantly associated with positive histology alone (OR 2.13; 0.81–5.6) compared to those without positive histology (Table 3).

When considering the combination of positive histology with positive microbiology, clinical failure rate was significantly higher (11.7% versus 5.5% for all other combinations (OR 2.30; 1.06–4.96)). When histology was positive, failure rate was not related to pathogen virulence within this subgroup analysis (11.8% virulent vs. 10.8% non-virulent (OR 1.11; 0.36–3.37) (Table 3).

## 4. Discussion

This is the largest study examining the association between microbiology and histology results and clinical outcomes in FRI. Although both positive microbiology and positive histology are independent diagnostic indicators of FRI in the Consensus guidelines [10,11], their relationship and combined association with outcome were unknown.

Achieving a microbiological diagnosis by culturing the pathogen is important for determining an effective antimicrobial regimen. However, many infected fractures are culture-negative, particularly when there is a chronic infected non-union or the patient has received prolonged broad-spectrum antibiotics prior to surgical sampling [27]. Culture negativity affected one quarter of our included patients, despite stopping antimicrobials two weeks before surgical sampling. In the present study, the nature of the microbiological culture did affect failure rate. Mixed Gram-positive and Gram-negative cultures had the highest failure rate (18.6%) followed by positive culture with virulent microorganisms (11.4%), non-virulent culture-positive FRI (6.9%) and culture-negative cases (5.5%). Furthermore, virulent infection was most likely to be associated with positive histology, particularly when *Staphylococcus aureus* was present, suggesting a link between pathogen type and host response.

**Figure 2:**
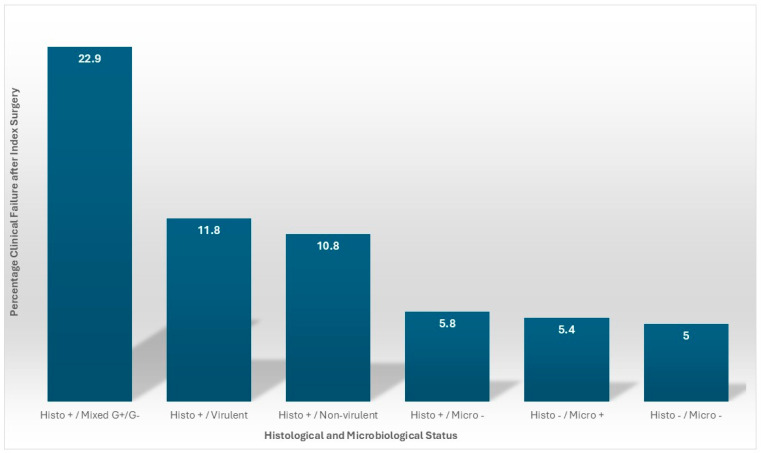
The relationship between microbiological and histological positivity and clinical outcome.

Our data showed that positive microbiology or histology, independently, were only weakly predictive of treatment failure. Additionally, when an inflammatory response was detectable in the tissues (positive histology), bacterial virulence alone did not predict clinical failure. This was well illustrated in the mixed Gram-positive and Gram-negative infections, where all of the clinical failures occurred in histology positive cases, with no failures when histology was negative. In combination, microbiology positive and histology positive cases resulted in a clear and significant increase in treatment failure. This suggests that it is not just the presence or nature of the bacteria at the site of the infection, but rather the presence of viable, culturable bacteria that elicit a host immune response, which dictates clinical outcome (Figure 2).

In a study of 110 unhealed fractures, 23 of 83 (28%) cases with no signs or symptoms of infection were found to have ‘low-grade’ infection on microbiological culture, usually with non-virulent organisms such as Coagulase-negative *Staphylococci* or *Cutibacterium acnes*. Only 22% of these cases had positive histology [37]. Interestingly, these 23 cases, with non-virulent infection and mostly negative histology, had a very low treatment failure rate, similar to cases without infection. This result mirrors our group of culture-negative and non-virulent bacteria with negative histology and better clinical outcome after surgical treatment.

One hypothesis for our findings is that when viable, culturable bacteria induce an inflammatory response, there is a higher risk of collateral tissue damage. This may serve to facilitate bacterial dispersion and opportunities for persistence, making surgical debridement more difficult and antimicrobials less effective, thereby increasing the risk of failure and relapse. This may be especially important with pathogens such as *Staphylococcus aureus*, which more frequently elicit a marked immune response and have multiple mechanisms for evasion of host defenses and tolerance of antimicrobials [20,38,39].

Increasingly, it is recognized that the host immune system plays a crucial role in bone metabolism and that immune dysregulation contributes to bone infection [24]. For example, inflammatory cytokines such as TNFα, IL-1, and IL-8 can increase osteoclastogenesis via RANKL production to enhance bone destruction, as well as inhibiting osteogenesis [40]. Specifically, *Staphylococcus aureus* can directly stimulate osteoclasts [41] and invade and survive within osteoblasts, disrupting their function [40]. In the broader context, our findings are consistent with the ‘damage response model’ of microbial pathogenesis [42]. This model suggests that the mechanism of disease is a spectrum, from damage caused directly by the pathogen to damage mediated through the host response to the infection. This concept has also been described in response to fungal pathogens [43]. Also, in a recent study, Jensen et al. showed that the neutrophilic response is strongly associated with tissue damage in chronic lung infections and that this response is modulated by the state of the bacteria (planktonic versus biofilm producing) [44]. Our findings, that positive histology (the presence of an inflammatory response) is indicative of a poor outcome is consistent with this hypothesis. It implies that clinical outcome after treatment is also on this spectrum. Simple colonization by a pathogen is more easily treated and has good outcomes. However, further along the spectrum, with polymicrobial infection and a prominent inflammatory response, the tissue damage may determine a poor outcome after surgery.

Our findings may indicate that histology positive FRI represents a neutrophil-rich, pro-inflammatory state. Osteoclasts can detect bacteria through MYD88 and activate the RANK pathway resulting in osteolysis [45]. However, infection-mediated bone loss is multifactorial, involving several components of the immune response [46].

In bone infection, excessive immune activation can change the tissue environment through the release of reactive oxygen species, proteases and pro-inflammatory cytokines which can in turn activate osteoclasts and suppress osteogenesis, thereby impairing bone healing [24]. This is particularly important in FRI, where successful outcome is a balance of fracture healing and infection eradication.

Conversely, microbiology positive cases without marked histological inflammation may reflect forms of persistence that are less dominated by neutrophil infiltration, which may include formation of biofilm or intra-cellular infections [47]. This may help explain why microbiological positivity alone was a weaker predictor of failure compared to combined histological and microbiological positivity. Experimental studies of chronic *S. aureus* infection have shown expansion of myeloid-derived suppressor cells and impairment of effector T-cell responses, mechanisms that may favor bacterial persistence and tolerogenic immunity [48].

Positive histology denotes the presence of neutrophils in response to the bacteria and, therefore, serves as a marker of septic inflammation. Neutrophils are considered effector cells of the acute inflammatory response, but due to pathogen persistence in FRI, they are continually recruited from the circulation to the infected fracture site, regardless of chronicity or infection duration. Neutrophils lead to extracellular matrix degradation by proteolytic enzymes, resulting in necrosis and tissue liquefaction of both osseous and non-osseous tissues. Furthermore, they release pro-inflammatory cytokines, which skew bone homeostasis towards pronounced osteoclast activation as previously mentioned. Indirectly, the neutrophils will also stimulate fibrosis at the fracture ends. This occurs due to both hypoxia and sustained chronic inflammation cytokine release, primarily from macrophages and lymphocytes, which will follow the neutrophil recruitment. These released cytokines are tailored to promote fibroblast proliferation and include, among others, fibroblast growth factor (FGF) and vascular endothelial growth factor (VEGF). More broadly, failure to transition from an early pro-inflammatory response toward a reparative immune environment may contribute to ongoing tissue injury and impaired healing in chronic bone infection [47], leading to a worse clinical outcome.

The role of host factors in the outcome of bone and joint infections has increasingly been recognized. Host complexity is represented in both the BACH [49] and FRI [50] classifications for osteomyelitis and FRI severity and has been shown to affect treatment outcome [27,51,52]. Chronic health conditions such as smoking, diabetes and obesity are thought to be associated with low-grade systemic inflammation [53,54], and so it may be that immune dysregulation is the unifying mechanism of poor outcome in this patient cohort too [55].

It is commonly accepted that the pathogen determines the timing and clinical presentation of infection in FRI (virulent bacteria causing early acute presentation, whereas low-grade pathogens present with indolent, chronic symptoms), although multiple groups have subsequently shown that this is not the case [56,57,58]. In fact, it may be pathogen-associated factors such as biofilm formation, antimicrobial resistance, and toxin production and their interaction with the host to produce inflammation that predicts clinical outcome, rather than the pathogen per se.

This study is limited by being a single center study and therefore results may not be generalizable. However, our conclusions are very similar to those from an independent Danish cohort studying paired sampling in FRI and osteomyelitis [27]. Further work on a larger cohort of patients would be useful, as well as further investigation of the mechanisms behind this complex relationship between osteoimmunology and bone infection.

In summary, clinical failure in FRI may be related to the presence of any viable, culturable bacteria, creating a measurable inflammatory response. Our data suggests that the host response to infection and the extent of inflammation are at least as important as the pathogen itself when predicting outcome in FRI.

## 5. Conclusions

This is the first study to demonstrate that outcome is less favorable in FRI cases where both microbiology and histology tests meet the diagnostic criteria for infection. This suggests a key role for the host inflammatory response to infection, rather than just the presence of viable microorganisms per se, in predicting clinical outcome. The mechanisms behind this interaction warrant further investigation.

## Figures and Tables

**Table 1:** Demographics, affected bone, culture type, and pathogen type with clinical outcome after surgical treatment.

	*n*	Success *n*	Fail *n* (%)	Odds Ratio (95% Confidence Interval)
All patients	430	390	40 (9.3)	
Male	322	294	28 (8.7)	1.31 (0.64–2.68) ^1^
Female	108	96	12 (11.1)	
Tibia	194	177	17 (8.8)	
Femur	111	100	11 (9.9)	
Ankle	40	36	4 (10.0)	
Upper limb	70	65	5 (7.1)	
Monomicrobial	229	209	20 (8.7)	8.86 (5.12–15.34) ^2^
Polymicrobial	92	78	14 (15.2)	14.55 (6.06–34.97) ^3^
Culture-negative	109	103	6 (5.5)	0.61 (0.24–1.56) ^4^
Gram-positive only	210	190	20 (9.5)	0.58 (0.17–2.04) ^5^ 0.42 (0.19–0.92) ^6^
Gram-negative only	52	49	3 (5.8)	
Mixed Gram-positive/negative	59	48	11 (18.6)	
*Staphylococcus aureus* isolated	169	150	19 (11.2)	0.86 (0.42–1.77) ^1^
Non-*Staphylococcus aureus* (but culture-positive)	152	137	15 (9.9)	

^1^ Non-significant difference at *p* < 0.05. ^2^ Monomicrobial vs. polymicrobial; significant difference at *p* < 0.05. ^3^ Culture-negative vs. polymicrobial; significant difference at *p* < 0.05. ^4^ Monomicrobial vs. culture-negative; non-significant difference at *p* < 0.05. ^5^ Gram-positive vs. Gram-negative; non-significant difference at *p* < 0.05. ^6^ Mixed culture vs. Gram-positive or Gram-negative culture; significant difference at *p* < 0.05.

**Table 2:** The relationship between pathogens and histological positivity.

Pathogen	*n*	Histology Positive	Histology Negative	% Positive
*Staphylococcus aureus*	170	152	18	89.4
*Staphylococcus epidermidis*	20	10	10	50.0
*Staphylococcus lugdunensis*	4	3	1	
Other CoNS ^1^	40	27	13	67.5
*Streptococcus* spp.	41	37	4	90.2
*Enterococcus* spp.	31	24	7	77.4
*Corynebacterium* spp.	12	11	1	91.7
*Cutibacterium acnes*	7	5	2	71.4
*Bacillus* spp.	2	2	0	
*Finegoldia magna*	3	2	1	
*Peptinophilus*	2	1	1	
*Anaerococcus*	1	1	0	
*Clostridium*	1	1	0	
*Escherichia coli*	31	28	3	90.3
Other *Escherichia*	1	1	0	
*Pseudomonas* spp.	31	24	7	77.4
*Enterobacter cloacae*	28	23	5	82.1
*Proteus* spp.	17	16	1	94.1
*Klebsiella* spp.	11	8	3	61.5
*Citrobacter* spp.	6	5	1	
*Serratia marcescens*	4	3	1	
*Bacteroides* spp.	5	4	1	
*Morganella morganii*	2	1	1	
*Aeromonas* spp.	2	2	0	
Mixed anaerobes	3	3	0	
*Halfnia alvei*	2	2	0	
*Leclercia*	1	1	0	
*Achromobacter*	1	1	0	
*Acinetobacter*	1	1	0	
Coliforms	1	1	0	
*Eikenella corrodens*	1	1	0	
*Burkholderia*	1	0	1	
*Campylobacter*	1	0	1	
Monomicrobial	229	189	40	82.5
Polymicrobial	92	76	16	82.6
Culture-negative	109	69	40	63.3
Gram-positive	334	276	58	82.6
Gram-negative	147	122	25	83.0
Gram-positive and Gram-negative	59	48	11	81.4
Virulent bacteria ^2^	390	336	54	86.2
Non-virulent bacteria	94	65	29	69.1

^1^ Coagulase-negative *Staphylococci*. ^2^ Virulent microorganisms were considered as uncommon contaminants, including: *Staphylococcus aureus*, *Staphylococcus lugdunensis*, beta-hemolytic *Streptococci*, *Streptococcus anginosus* group, *Enterococci* spp., *Enterobacterales*, *Pseudomonas aeruginosa*, and anaerobic Gram-negative rods [25].

**Table 3:** The relationship between microbiological and histological positivity and clinical outcome after surgical treatment.

	*n*	Success *n*	Fail *n* (%)	Odds Ratio (95% Confidence Interval)
All patients	430	390	40 (9.3)	
Microbiology positive	321	287	34 (10.6)	2.03 (0.83–4.96) ^1^
Culture-negative	109	103	6 (5.5)	
Histology positive	334	299	35 (10.5)	2.13 (0.81–5.60) ^1^
Histology negative	96	91	5 (5.2)	
Virulent pathogen isolated	265	238	27 (10.2)	1.08 (0.42–2.75) ^1^
Non-virulent pathogens only	55	49	6 (10.9)	
Histology positive/VP ^2^	228	201	27 (11.8)	1.11 (0.36–3.37) ^1^
Histology positive/N-VP ^3^	37	33	4 (10.8)	
Microbiology and histology positive	265	234	31 (11.7)	2.30 (1.06–4.96) ^4^
Microbiology and/or histology negative	165	156	9 (5.5)	

^1^ Non-significant difference at *p* < 0.05. ^2^ VP; virulent pathogen cultured. ^3^ N-VP; non-virulent pathogen cultured. ^4^ Significant differences at *p* < 0.05.

## Data Availability

The original contributions presented in this study are included in the article. Further inquiries can be directed to the corresponding author.

## Abbreviations

The following abbreviations are used in this manuscript:

FRI	Fracture-related infection
TNFα	Tumor necrosis factor alpha
CoNS	Coagulase-negative Staphylococci
RANKL	Receptor Activator of Nuclear Factor kappa-B Ligand
IL-1	Interleukin 1
IL-8	Interleukin 8
